# Early Ultrasound Assessment of Renal Transplantation as the Valuable Biomarker of Long Lasting Graft Survival: A Cross-Sectional Study

**DOI:** 10.5812/iranjradiol.11492

**Published:** 2014-01-30

**Authors:** Ahmad Enhesari, Saeid Mardpour, Zohreh Makki, Soura Mardpour

**Affiliations:** 1 Department of Radiology, Faculty of Medicine and Health Sciences, Kerman University of Medical Sciences, Kerman, Iran; 2 Applied Cell Sciences Department, School of Advanced Technologies in Medicine, Tehran University of Medical Sciences, Tehran, Iran

**Keywords:** Kidney Transplantation, Ultrasonography, Glomerular Filtration Rate

## Abstract

**Background:**

To date, there has been little agreement on the use of ultrasonographic parameters in predicting the long-term outcome after transplantation. This study evaluates whether ultrasonography of the graft performed in the early stage after transplantation is a valuable predictor for long-term-outcome.

**Objectives:**

The aim of this study was to evaluate the association of ultrasonographic parameters (resistive index [RI], pulsatility index[PI], end diastolic velocity [EDV], graft length and graft parenchymal volume) measured within the first week after transplantation with 6 months graft function.

**Patients and Methods:**

A cross-sectional study was performed on 91 (46 males and 45 females) living renal transplants between April 2011 and February 2013. All patients underwent an ultrasonography at the first week after transplantation. Intrarenal Doppler indices including RI, PI and EDV were measured at the interlobar artery level and the graft length and parenchymal volume were defined with gray scale ultrasonography. Graft function was estimated at 6months by glomerular filtration rate (GFR). Unpaired t-test and multivariate-linear and logistic regression analysis were used to estimate the relationship between ultrasonographic parameters and GFR.

**Results:**

Fourteen patients (15.4%) had impaired graft function after 6 months (GFR less than 60 ml/min/1.73m2). Multivariate linear regression analysis showed significant correlation between GFR at 6 months and RI, PI and EDV with a P value of 0.026, 0.016 and 0.015, respectively. Logistic regression analysis showed that GFR<60 ml/min/1.73 m2 at 6 months was significantly associated with RI>0.7 (odds ratio=2.20, P value=0.004) and PI>1.3 (odds ratio=2.74, P value<0.001) and EDV<9 cm/Sec (odds ratio=1.83, P value=0.03).

**Conclusions:**

In this study, kidney transplant recipients with a lower RI and PI and a higher EDV at 1week showed better graft function at 6 months after transplantation.

## 1. Background

Ultrasound is a noninvasive and relatively inexpensive diagnostic tool providing information about renal location, contour and size. Doppler ultrasonography shows kidney morphology and hemodynamics (1). It is widely used to evaluate the graft complications such as obstruction, perirenal collection or vascular complications such as rejection or renal arterial/venous thrombosis (2, 3).

Resistive index (RI) has been shown as the best ultrasonographic parameter to determine renal dysfunction (4, 5). It is used as a marker of microcirculation injury and a sequel of interstitial edema of any etiology (6, 7). Other parameters such as the pulsatility index (PI) and graft dimensions are also used in this circumstance. It was believed that ultrasonography is used just for discrimination of acute rejection episodes or other complications but in recent years, literatures have shown that RI and some other ultrasonographic parameters are handled to predict long-term graft function. These parameters consist of RI, PI, end diastolic velocity (EDV) and graft length. RI with a normal value immediately after transplantation is a good predictor of the future graft function. RI not only reflects resistance at the arterial blood flow of renal arteries, but also shows proximal pathology such as systemic blood pressure (8).

Intrarenal diastolic blood flow has no association with systolic components; therefore, the end diastolic velocity (EDV) obtained with Doppler wave tracing illustrates pathologic changes at kidney graft more reliably than RI (8, 9).

PI is another Doppler parameter more emphasized on Doppler wave pattern and has a significant value in graft function prediction. On the other hand, some gray scale parameters such as graft size are often used to predict long-term graft function (10).

## 2. Objectives

The aim of this study was to evaluate the association of ultrasonographic parameters (RI, PI, EDV, graft length and graft parenchymal volume) measured within the first week after transplantation with 6 months graft function.

## 3. Patients and Methods

Between April 2011 and February 2013, a total of 100 patients who underwent renal transplantation in Afzalipour university hospital were enrolled and a single investigator performed all ultrasonographies by MEDISON V10 device (ACCUVIX V10, MEDISON Co. LTD, Korea) with a curved probe of 3.5-5 MHz (C3-7IM) in the supine position. We consulted the local ethics committee regarding this study. No formal ethical committee approval was required.

**Figure 1.**
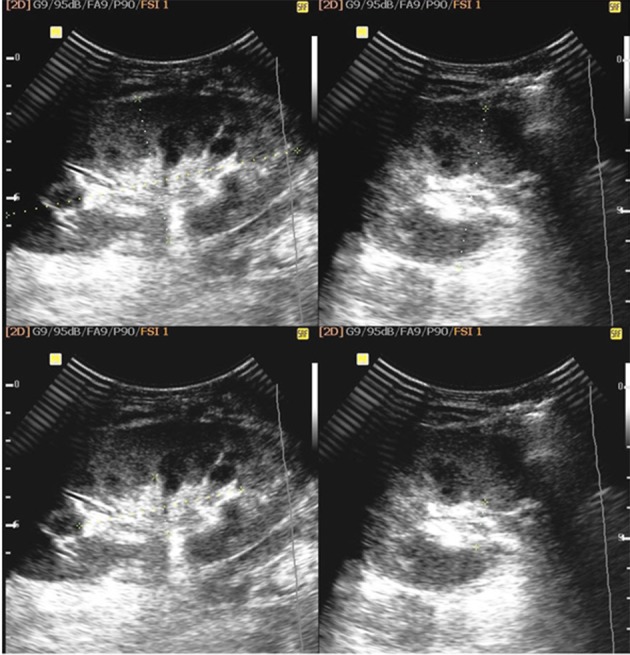
Graft length and graft parenchymal volume measurement by gray scale sonography

Three patients died and six patients were excluded from the study owing to factors that influenced the Doppler parameters; three patients due to hydronephrosis, two patients due to arterial stenosis and one patient due to perirenal collection (1, 2). The level of creatinine was measured daily up to a stable level for each patient. All patients were assessed by ultrasonography at approximately the first week after transplantation when the creatinine level was normal (below 1.5 mg/dl) and kidney graft size and parenchymal volume (renal volume – renal sinus volume) were determined (Figure 1). 

**Figure 2.**
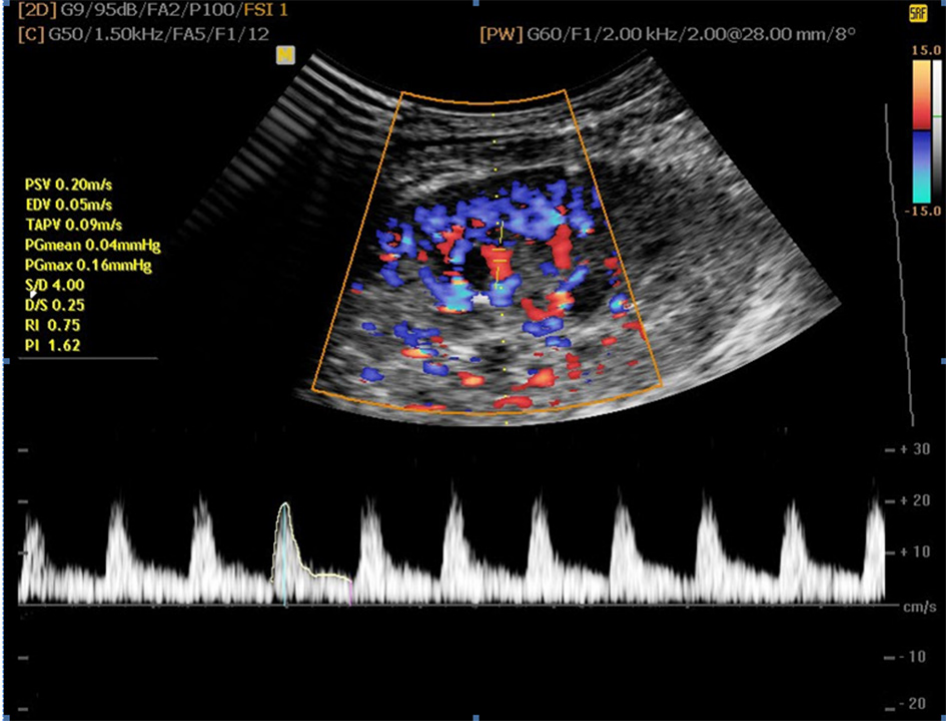
RI, PI and EDV assessment by Doppler sonography

RI and PI were calculated by system software at an interlobar artery level (Figure 2) according to the equations:

RI=(V max – V min)/V max

PI=(V max – V min)/V mean

V max is the maximum systolic velocity, V min is the minimum diastolic velocity, and V mean is the time-averaged mean velocity (11).

EDV was calculated by system software. The renal function was evaluated by measurement of serum creatinine (Cr) and estimation of the gloumerular filtration rate (GFR) according to the equation:

$$\mathrm{GFR}=\frac{(140-\mathrm{age})\times \text{ body weight (multiplied by 0.85 in females)}}{\mathrm{Cr}\times 72}$$

Age was measured in year, body weight in Kg, creatinine in mg/dl, GFR in ml/min/per 1.73 m^2^ and EDV in cm/Sec.

Patients were classified as display decreased graft function when GFR was <60 and normal graft function when GFR was ≥60. Continuous variables were expressed as a mean value±standard deviation. The differences between patient groups were assessed with unpaired standard t-test. The degree of correlation between ultrasonographic parameters and GFR were estimated by multi linear regression models. Logistic regression analysis was used to estimate the potential association between ultrasound parameters and decreased graft function versus near normal graft function at 6 months post transplantation. All statistical tests were interpreted as two tailed. P values lower than 0.05 were considered as statistically significant. Analyses were performed using SPSS (IBM Corp. Released 2012. IBM SPSS Statistics for Windows, Version 21.0. Armonk, NY: IBM Corp).

## 4. Results

Of the 91 patients with the mean age of 36.9±10.7 years (range, 14-69 years), 46 (50.6%) were male and 45 (49.4%) were female. They were followed up for 6 months after transplantation. Fourteen patients (15.4%) had impaired graft function after 6 months (GFR less than 60 ml/min/1.73 m^2^). The median of RI and PI at the first week after grafting was 0.71 and 1.2, respectively. Mean RIs were 0.68±0.07 and 0.79±0.07 in patients with normal graft function and graft dysfunction at 6 months after transplantation, respectively (Table 1). On the other hand, the mean PIs at 6 months after transplantation in patients with stable graft function and graft dysfunction were 1.17±0.25 and 1.7±0.54, respectively and at the same condition; the mean EDVs were 9.46±3.6 and 6.6±2.9, respectively (Table 1). This means that patients with stable graft function at 6 months had a lower RI and PI and a higher EDV. Independent t-test showed significant differences between mean RI, mean PI and mean EDV of patients with 6 months normal and impaired graft function (P value<0.001, P value<0.001 and P value=0.002 for RI, PI and EDV, respectively). Both groups neither demonstrated a difference in length (P value=0.801) nor parenchymal volume (P value=0.617) (Table 1). Multivariate linear regression analysis showed a significant correlation between GFR at 6 months with RI (P value=0.026), PI (P value=0.016) and EDV (P value=0.015) during 1st week post transplantation. No association between graft length, renal parenchymal volume and graft future function were obtained (P values=0.668 and 0.56 respectively). Logistic regression analysis demonstrated a significantly greater odds ratio for decreased graft function at 6 months post transplantation among patients with RI>0.7 (Odds ratio=2.20), PI>1.3 (Odds ratio=2.74) and EDV<9 cm/Sec (Odds Ratio=2.1) (Table 2). 

**Table 1.** Mean Value of Parameters Measured at the First Week after Transplantation According to the GFR Estimated at 6 Months Post Transplantation ^a^

Values	GFR≥60 (n=77)	GFR<60 (n=14)	P value (Independent t-test)
**RI**	0.68±0.07	0.79±0.07	<0.001
**PI**	1.17±0.25	1.7±0.54	<0.001
**EDV**	9.46±3.6	6.6±2.9	0.002
**Parenchymal Volume**	138±37.6	143±38.3	0.617
**Kidney Length**	106±9.3	107±9.3	0.801

^a^ Abbreviations: RI; resistive index, PI; pulsatily index, GFR; glumerular filtration Rate, EDV; end diastolic velocity

**Table 2.** Correlation Between Parameters of Kidney Graft with the GFR Estimated at 6 Months After Transplantation Assessed by Logistic Regression Analysis

Value	Odd Ratio	P Value
**RI**	2.20	0.004
**PI**	2.74	<0.001
**EDV**	2.1	0.03

## 5. Discussion

Ultrasonographic parameters are widely used to evaluate not only the present graft function, but also as predictive factors of long term outcome of the renal transplant. These parameters have been shown to correlate with short-term renal transplant function determined by serum creatinine or GFR. However, their relationship with long-term function is more controversial, with conflicting results in the literature (9).

The results of this study indicate that RI, PI and EDV in the early phase after transplantation are significantly associated with renal dysfunction 6 months after transplantation. Intrarenal RI is influenced by not only arterial resistance but also various extararenal factors such as the systemic blood pressure. Therefore, RI parameter as a sole element in distinguishing various causes of graft dysfunction has a limited value. On the other hand, an increase in RI can be induced by any interarenal condition such as acute renal failure or urinary tract obstruction that induces a reduction in diastolic renal perfusion (10, 11). PI is altered by both physiologic and pathologic conditions. The status of cardiac function and systemic circulation and flow resistance are important factors influenced by PI. Unlike the RI, whole wave shape at one cardiac cycle is calculated by PI and therefore, the PI is a better indicator of graft function than the RI (12). Furthermore, interarenal diastolic flow reflects high vascular resistance within the renal allograft circulation more specifically than RI (8). EDV is measured simply and if it is measured parallel to RI parameter, it provides more specific information about renal resistance to flow. Low diastolic flow is an indicator of poor prognosis for graft survival. Previous studies have shown a significant correlation between long-term graft function and RI, PI and EDV.

Radermacher et al. (5) showed that RI is the best predictor for graft failure. McArthur et al. (10) demonstrated that assessment of PI and RI in the early post transplantation period are significantly associated with long-term transplant outcome including 1-year GFR and transplant survival.

Buturovic-Ponikvar et al. (9) demonstrated that RI, EDV and graft length have a predictive value in estimating long-term graft function. Barba et al. (13) found that RI measured as early as 24hr after surgery can predict the long-term graft survival. Adibi et al. (14) showed that early determination of RI and PI could predict long-term graft function in kidney transplant recipients. Kramann et al. (15) published a paper in which they described that only RIs evaluated between 12-18 months after transplantation can predict long-term graft function.

On the other hand, some researchers did not support this correlation. Loock et al. (16) and Garcia-Covarrubias et al. (17) have shown no correlation between future graft function and numerous parameters of kidney ultrasonography. Like most of the previous studies, we found that RI, PI and EDV measurement within the first week of grafting significantly correlate with graft function at 6 months follow-up. Interestingly, transplant recipients suffering higher RI and lower EDV and especially higher PI at early stages, showed impaired graft function at 6 months aftertransplantation. This means that RI>0.7, PI>1.3 and EDV<9 cm/Sec could be considered as an indicator of not only vascular complications but also poor outcome of the graft. Buturovic-Ponikvar et al. (9) observed that the graft size before transplantation is significantly associated with creatinine clearance at 12 months. This experiment did not detect any correlation between renal parenchymal volume, graft length and graft future function. It seems possible that the results of Buturovic-Ponikvar were due to the kidney size measurement by weighing the graft before transplantation but we have checked it after transplantation and it had significant correlation with the long-term graft function.

In recent years, management of renal transplant recipients has progressively improved. Moreover, it is necessary to use available short-term tools to predict long-term graft function. Applying these factors make better management of the transplants. In spite of the controversies regarding ultrasound assessment in kidney transplants, it is considered as a prognostic marker for long-term graft survival. This study focused on evaluating whether ultrasonography performed in the early period after transplantation would be a valuable predictor of long-term-outcomes.

Our findings demonstrate that out of the different ultrasonographic parameters that were measured within the first week after renal transplantation, only the early evaluation of RI, PI and EDV were effective in the estimation of long-term graft function.
